# The impact of a secondary, rare, non-pathogenic *PKD1* variant on disease progression in autosomal dominant polycystic kidney disease

**DOI:** 10.1007/s40620-025-02211-x

**Published:** 2025-01-30

**Authors:** Elhussein A. E. Elhassan, Kane E. Collins, Sophia Heneghan, Edmund Gilbert, Hana Yang, Sarah R. Senum, Rachel S. Schauer, Doaa E. Elbarougy, Stephen F. Madden, Susan L. Murray, Omid Sadeghi-Alavijeh, Joshua Carmichael, Daniel Gale, Shohdan M. Osman, Claire Kennedy, Matthew D. Griffin, Liam Casserly, Brona Moloney, Paul O’Hara, Amali Mallawaarachchi, Francesca Ciurli, Genomics England Consortium, John C. Ambrose, Prabhu Arumugam, Marta Bleda, Freya Boardman-Pretty, Christopher R. Boustred, Helen Brittain, Mark J. Caulfield, Georgia C. Chan, Tom Fowler, Adam Giess, Angela Hamblin, Shirley Henderson, Tim J. P. Hubbard, Rob Jackson, Louise J. Jones, Dalia Kasperaviciute, Melis Kayikci, Athanasios Kousathanas, Lea Lahnstein, Sarah E. A. Leigh, Ivonne U. S. Leong, Javier F. Lopez, Fiona Maleady-Crowe, Loukas Moutsianas, Michael Mueller, Nirupa Murugaesu, Anna C. Need, Peter O’Donovan, Chris A. Odhams, Christine Patch, Daniel Perez-Gil, Mariana B. Pereira, John Pullinger, Tahrima Rahim, Augusto Rendon, Tim Rogers, Kevin Savage, Kushmita Sawant, Richard H. Scott, Afshan Siddiq, Alexander Sieghart, Samuel C. Smith, Alona Sosinsky, Alexander Stuckey, Melanie Tanguy, Ellen R. A. Thomas, Simon R. Thompson, Arianna Tucci, Emma Walsh, Matthew J. Welland, Eleanor Williams, Katarzyna Witkowska, Suzanne M. Wood, Claudio Graziano, Constantin A. Wolff, Ria Schönauer, Gaetano LaManna, Axelle Durand, Sophie Limou, Jan Halbritter, Irene Capelli, Emma McCann, Peter C. Harris, Gianpiero L. Cavalleri, Katherine A. Benson, Peter J. Conlon

**Affiliations:** 1 Department of Nephrology, Beaumont Hospital, Dublin, Ireland; 2 Department of Medicine, Royal College of Surgeons in Ireland, Dublin, Ireland; 3 School of Pharmacy and Biomolecular Sciences, Royal College of Surgeons in Ireland, Dublin, Ireland; 4 The Science Foundation Ireland FutureNeuro Centre of Excellence, Dublin, Ireland; 5 SFI Research Ireland Centre for Research Training in Genomics Data Science, University of Galway, Galway, Ireland; 6 Division of Nephrology & Hypertension, Department of Internal Medicine, Mayo Clinic, Rochester, MN USA; 7 Data Science Centre, Royal College of Surgeons in Ireland, Dublin, Ireland; 8 Department of Renal Medicine, University College London, London, UK; 9 Genomics England, Queen Mary University of London, London, UK; 10 Kingston General Hospital & Queen’s University, Kingston, ON Canada; 11 Regenerative Medicine Institute (REMEDI) at CÚRAM Centre for Research in Medical Devices, School of Medicine, University of Galway, Galway, Ireland; 12 Nephrology Department, Galway University Hospitals, Saolta University Healthcare Group, Galway, Ireland; 13 Department of Renal Medicine, University Hospital Limerick, Limerick, Ireland; 14 Rare Disease Program, Garvan Institute of Medical Research, Sydney, NSW Australia; 15 Department of Medical Genomics, Royal Prince Alfred Hospital, Sydney, NSW Australia; 16 Nephrology, Dialysis and Kidney Transplant Unit, IRCCS Azienda Ospedaliero-Universitaria di Bologna, 40138 Bologna, Italy; 17 Medical Genetics Unit, AUSL Romagna, 47522 Cesena, Italy; 18 Department of Nephrology and Medical Intensive Care, Charité-Universitätsmedizin Berlin, Berlin, Germany; 19 Division of Nephrology, Department of Endocrinology, Nephrology, and Rheumatology, University of Leipzig Medical Center, Leipzig, Germany; 20 Department of Medical and Surgical Sciences (DIMEC), Alma Mater Studiorum University of Bologna, 40138 Bologna, Italy; 21 Ecole Centrale Nantes, INSERM, Center for Research in Transplantation and Translational Immunology, UMR1064, Nantes University, Nantes, France; 22 The Department of Clinical Genetics, Children’s Health Ireland at Crumlin, Dublin, Ireland

**Keywords:** Genetic burden, PKD, ADPKD, Disease severity, Prognosis

## Abstract

**Background:**

Autosomal dominant polycystic kidney disease (ADPKD) is caused primarily by pathogenic variants in the *PKD1* and *PKD2* genes. Although the type of ADPKD variant can influence disease severity, rare, hypomorphic *PKD1* variants have also been reported to modify disease severity or cause biallelic ADPKD. This study examines whether rare, additional, potentially protein-altering, non-pathogenic *PKD1* variants contribute to ADPKD phenotypic outcomes.

**Methods:**

We investigated the prevalence of rare, additional, potentially protein-altering *PKD1* variants in patients with *PKD1*-associated ADPKD*.* The association between rare, additional, potentially protein-altering variants and phenotypic outcomes, including progression to kidney failure, age at onset of hypertension and urological events, height-adjusted total kidney volume, and predicting renal outcomes in PKD (PROPKD) score, were examined.

**Results:**

Rare, additional, potentially protein-altering variants were detected in 6% of the 932 ADPKD patients in the study. The presence of rare, additional, potentially protein-altering variants was associated with 4 years earlier progression to kidney failure (hazard ratio (HR): 1.66; 95% confidence interval (CI): 1.18–2.34; *P* = 0.003), with in-*trans* rare, additional, potentially protein-altering variants (*n* = 13/894) showing a greater risk of kidney failure (HR: 1.83; 95% CI 1.00–3.33; *P* = 0.049). We did not detect statistically significant differences between rare, additional, potentially protein-altering variants and other phenotypic outcomes compared to those without rare, additional, potentially protein-altering variants.

**Conclusions:**

In patients with *PKD1*-associated ADPKD, our findings suggest that rare, additional, potentially protein-altering variants in *PKD1* may influence disease severity. These findings have potential clinical implications in counselling and treating patients with rare, additional, potentially protein-altering variants, but further investigation of such variants in larger, longitudinal cohorts with detailed, standardised phenotype data is required.

**Graphical abstract:**

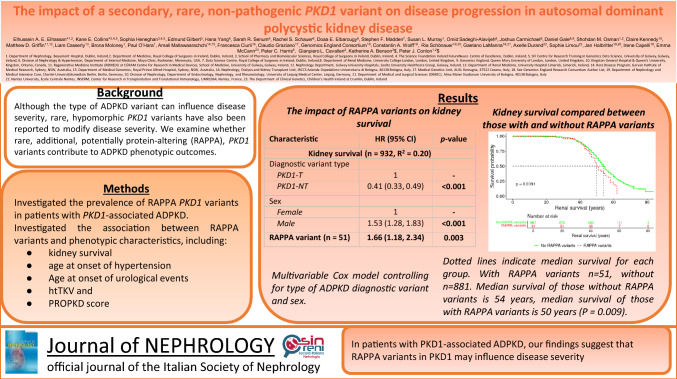

**Supplementary Information:**

The online version contains supplementary material available at 10.1007/s40620-025-02211-x.

## Plain Language Summary

Autosomal dominant polycystic kidney disease (ADPKD) is usually caused by variants in the *PKD1* and *PKD2* genes. This study found that people with *PKD1* ADPKD carrying additional damaging genetic variants in the *PKD1* gene, progress to kidney failure at an earlier age.

**Supplementary Information:**

The online version contains supplementary material available at 10.1007/s40620-025-02211-x.

## Key Points

**Table:** 

In ADPKD patients with a confirmed genetic diagnosis, additional rare, non-pathogenic, but inferred damaging *PKD1* variants result in an increased risk and an earlier age of kidney failure compared to those that did not carry such additional *PKD1* variants.	

## Introduction

Autosomal dominant polycystic kidney disease (ADPKD) is the most common Mendelian kidney disease and may result in end-stage kidney disease[1]. ADPKD is a genetically and phenotypically heterogeneous disorder, with approximately 80–85% of cases caused by variants in *PKD1*, and 15–20% by variants in *PKD2*. Recently, monoallelic *IFT140* variants, and rare heterozygous variants in *GANAB*, *DNAJB11*, *ALG8*, *ALG9*, and *ALG5* were shown to cause milder ADPKD-spectrum disorders[2].

ADPKD typically manifests in adulthood with bilateral cystic kidneys. The disease course is often asymptomatic at an early age, but systemic manifestations, such as hypertension, abdominal pain, haematuria, urinary tract infections, and cerebral aneurysms may be evident before a decline in the glomerular filtration rate (GFR) becomes apparent[3]. As the kidney enlarges, GFR declines due to the destruction of the renal parenchyma caused by cystic dilation, commonly progressing to kidney failure. However, the rate of disease progression is variable; around half of the affected individuals develop kidney failure by their sixth decade[4]. It is well established that the pathogenic ADPKD gene can influence disease severity[5]. Patients with pathogenic *PKD1* variants have a more severe form of disease with faster rates of progression to kidney failure than patients with pathogenic *PKD2* variants[1, 5]. Further, *PKD1* truncating (*PKD1*-T) variants, on average, cause a more severe form of the disease than *PKD1* non-truncating (*PKD1*-NT) variants[5]*.*

Protein truncating variations in *PKD1* and *PKD2* are understood to be largely fully penetrant[1]. However, it has been suggested that specific missense variants in *PKD1* and *PKD2* that do not cause disease in isolation, may cause very early onset disease when two alleles are inherited *in trans*[6–9]. The impact of additional variants *in trans* to the diagnostic variant has also been demonstrated in inherited retinal diseases[10]. Further, in case studies of genetically resolved ADPKD pedigrees, wide variability in disease severity has been reported in patients who also harbour these milder, non-pathogenic ADPKD variants[11].

Functional investigations support the potential disease-modifying effect of milder ADPKD variants. Mice heterozygous for the established hypomorphic allele *PKD1* NM_001009944:p.(R3277C) are phenotypically normal, while mice homozygous for this variant gradually develop cystogenesis[12]. Mice of different genetic backgrounds but homozygous for NM_001009944:p.(R3277C) have mild but progressive disease with significantly different levels of cyst development[12].

As different aspects of phenotypic and genetic heterogeneity in ADPKD are likely due to additional genetic and environmental factors[6], we hypothesised that rare variant burden in the form of rare, additional, potentially protein-altering (RAPPA) variants in *PKD1* may contribute to the spectrum of phenotypic variability seen in ADPKD. We tested this hypothesis in a cohort of 932 patients with a diagnosis of ADPKD due to a diagnostic variant (including likely pathogenic) in *PKD1*, assembled across seven international centres.

## Materials and methods

All participants provided informed consent using protocols approved by the relevant ethics committees or institutional review boards (see Supplementary Material). Participants were recruited from seven sites: Dublin-Ireland (Beaumont Hospital), Leipzig-Germany (University of Leipzig Medical Center), Bologna-Italy (Alma Mater Studiorum, University of Bologna), Sydney-Australia (Garvan Institute of Medical Research), Nantes-France (Nantes University Hospital), Rochester-United States (Mayo Clinic), and London-United Kingdom (Genomics England). To be eligible for inclusion, a clinical diagnosis of multiple kidney cysts on imaging and the confirmation of *PKD1-*associated ADPKD, detected using next-generation or Sanger sequencing methods, were required.

With the exception of the Nantes cohort, analysis of *PKD1* was conducted using next generation (targeted panel, whole exome, or whole genome sequencing) or Sanger sequencing data. All cohorts, with the exception of Nantes, have been published, at least in part (see Supplementary Material). The Nantes cohort was fully sequenced using whole exome sequencing and was unpublished. Progression to end-stage kidney disease was ascertained for each patient, determined by the initiation of chronic dialysis or pre-emptive kidney transplantation. Total kidney volume (TKV) was determined using manual segmentation or automated analysis of magnetic resonance or computed tomography data[13]. Height-adjusted TKV (htTKV) was calculated by dividing by patient height in metres. Hypertension status was defined as blood pressure in excess of 140/90 mmHg. Age at onset of hypertension was defined as age at diagnosis of hypertension or commencement of antihypertensive medications. Age at first urological event was defined as age at diagnosis of the first episode of either flank pain or macroscopic haematuria or kidney cyst infection. Mayo Imaging Class (MIC) and Predicting Renal Outcome in PKD (PROPKD) scores were also calculated. Diagnostic (pathogenic and likely pathogenic) variants were determined locally by the contributing centres, using the American College of Medical Genetics and Genomics (ACMG) guidelines[14] and expert opinion.

*Rare, additional, potentially protein-altering variants filtration* Rare aggregate genetic variations in *PKD1*, referred to hereafter as rare, additional, potentially protein-altering variants, were variants that satisfied all of the following six criteria:

(i) within the *PKD1* gene (NM_001009944.3),
(ii) with minor allele frequency (MAF) of < 1% in both 1) the global gnomAD v4.0 dataset and 2) all individual gnomAD “ancestry groups”[15],
(iii) within coding regions (not including splicing and synonymous variants),
(iv) predicted damaging by either PolyPhen2 or SIFT if the variant is missense (excluding missense variants predicted as “T” by SIFT (< 0·05) and “B” by PolyPhen2 (< = 0·452),
(v) have either a combined annotation-dependent depletion (CADD) (version 1.7) score[16] > 20, or rare exome variant ensemble learner (REVEL) score[17] > 0.5. Note that a combined annotation-dependent depletion score > 20 corresponds to the top 1% of all predicted most deleterious variants in the genome[16]. A rare exome variant ensemble learner score > 0.5 corresponds to a sensitivity of 0.75 and a specificity of 0.89 for predicting pathogenic variants[17],
(vi) be classified as a variant of unknown significance (VUS) using ACMG guidelines.

To identify rare, additional, potentially protein-altering *PKD1* variants, each centre re-examined the sequencing data of their genetically-confirmed ADPKD patients using the specified criteria, while all subsequent analyses were performed centrally in Dublin, Ireland. Note that rare, additional, potentially protein-altering variants are a subset of ACMG “variants of unknown significance”.

*Phasing variants* Phasing data was derived based on a combination of familial information and computational phasing analysis. Computational phasing analysis was carried out using SHAPEIT5 software[18] on the 9 individuals with rare, additional, potentially protein-altering variants in the Dublin cohort for whom genotyping data were available, and so could not be phased. Phasing data were also obtained for a further five individuals based on familial data. A common phasing scaffold was created using single nucleotide polymorphisms (SNPs) with minor allele frequency > 0.01 using the *phase_common* tool, and the rest of the single nucleotide polymorphisms were phased onto the common scaffold using the *phase_rare* tool.

*Accounting for familial structure* Of the 932 individuals in this dataset, there are 747 unique families. To demonstrate that the results were not confounded by familial relatedness, one individual from each family was randomly selected and retained while all other family members were removed. The Cox model was then rerun using this set of 747 unrelated individuals and compared to the results from the full cohort of 932 individuals.

*Impact of trans rare, additional, potentially protein-altering variants on kidney survival* Based on the computational phasing data, and the familial phasing data where either were available, individuals were classified as having rare, additional, potentially protein-altering variants that were either unknown (31/51), *in trans* (13/51) or *in cis* (7/51) of the primary ADPKD diagnostic variant. Individuals with unknown phasing were then excluded and a Cox model was created for kidney survival, taking covariates of sex, diagnostic variant type, and *in trans* rare, additional, potentially protein-altering variant. Similarly, a model was also created for the *in cis* rare, additional, potentially protein-altering variants compared to those without any rare, additional, potentially protein-altering variants.

Association of rare, additional, potentially protein-altering variants with outcomes of interest: All analyses were conducted in R, using version 4.2.1 (2022–06-23). A multivariable Cox regression proportional hazards model was used to establish the effect of rare, additional, potentially protein-altering variants on kidney survival, which adjusted for the diagnostic variant type (*PKD1*-T or *PKD1*-NT) and sex as covariates. A multivariable Cox model was also created for kidney survival, controlling for diagnostic variant and sex. A Kaplan–Meier plot was also generated, using the R function *ggsurvplot*, comparing kidney survival between individuals with rare, additional, potentially protein-altering variants and those without. Cox models were constructed to investigate the association between rare, additional, potentially protein-altering variants with both age at hypertension diagnosis and age at onset of urological events. The variance explained by each of the models (*R*^2^) was calculated. The assumptions of a Cox model (proportional hazards, nonlinearity and influential observations) were checked.

A multivariable linear model for the log transformed htTKV was constructed, controlling for age at imaging, diagnostic variant type, and sex. htTKV was log transformed to ensure it was normally distributed. MIC score was transformed from class 1A-1E to numeric values of 1–5. Univariable linear models to assess the association between rare, additional, potentially protein-altering variants and (1) MIC score and (2) PROPKD score were constructed. Assumptions of linear models (residuals vs fitted, normal Q-Q, scale-location, and residuals vs leverage) were checked.

## Results

### Clinical description of the study cohort

The total cohort consisted of 932 ADPKD patients with a diagnostic variant in *PKD1*. A preponderance of females was observed, comprising 59% (553) of the patient population, and 495 patients (53%) had reached kidney failure, at a median age of 53 years (Table 1). For all the other clinical outcomes of interest, there was a significant amount of missing data, with some centres unable to provide data on particular outcomes. htTKV data were available for 41% (*n* = 379/932) of the cohort, while age at onset of hypertension and age at onset of urological events were available for 34% (*n* = 316/932) and 35% (*n* = 323/932) of the cohort, respectively. MIC and PROPKD scores were both available for 41% (*n* = 382/932) and 35% (*n* = 328/932) of the cohort, respectively.

**Table 1:** Summary data for all cohorts. “Unknown” refers to the number of patients with missing data

	Overall	Bologna	Dublin	Leipzig	London	Nantes	Rochester	Sydney
Number of patients	932	74	304	29	103	40	357	25
Sex (Female), n (%)	553 (59)	38 (51)	172 (57)	11 (38)	65 (63)	25 (62)	230 (64)	12 (48)
Diagnostic variant type, *n* (%)								
PKD1-T	594 (64)	54 (73)	204 (67)	18 (62)	78 (76)	25 (62)	195 (54)	20 (80)
PKD1-NT	339 (36)	20 (27)	100 (33)	11 (38)	25 (24)	15 (38)	163 (46)	5 (20)
RAPPA variants^a^, *n* (%)	51 (6)	4 (5)	14 (5)	8 (28)	9 (9)	2 (5)	11 (3)	3 (12)
Kidney failure, *n* (%)	495 (53)	47 (64)	203 (67)	12 (41)	23 (22)	35 (88)	156 (44)	19 (76)
Age at kidney failure (years), median (range)	54 (15–79)	53 (32–79)	51 (15–79)	58 (36–61)	60 (29–68)	50 (31–69)	57 (19–75)	50 (30–64)
htTKV (mL/m), median (range)	1139 (180–13,110)	1,187 (237–3,496)	1471 (241–5,909)	1032 (225–2,658)	NA	NA	955 (180–13,110)	NA
Unknown	553 (59)	55 (74)	196 (64)	15 (52)	103 (100)	40 (100)	119 (33)	25 (100)
MIC, median	3	4	3	3	NA	NA	3	NA
Unknown	550 (59)	54 (73)	196 (64)	13 (45)	103 (100)	40 (100)	119 (33)	25 (100)
PROPKD score, median	5	5	6	5	NA	NA	NA	NA
Unknown	604 (65)	56 (76)	22 (7)	1 (3)	103 (100)	40 (100)	357 (100)	25 (100)
Age at first urological event (years), median (range)	40 (2–75)	53 (15–74)	38 (2–75)	46 (28–77)	NA	NA	NA	NA
Unknown	609 (65)	41 (55)	23 (8)	20 (69)	103 (100)	40 (100)	357 (100)	25 (100)
Age at hypertension diagnosis (years), median (range)	36 (15–77)	43 (15–60)	35 (15–77)	50 (28–77)	NA	NA	NA	NA
Unknown	616 (66)	48 (65)	20 (7)	23 (79)	103 (100)	40 (100)	357 (100)	25 (100)

*PKD1*-NT *PKD1* non-truncating variants, *PKD1*-T *PKD1* truncating variants, *RAPPA* rare, additional, potentially protein altering variants, *n* number, *CI* confidence interval, *MIC* Mayo imaging classification, *PROPKD* predicting renal outcomes in PKD

^a^Of the 51 individuals who have RAPPA variants, 3 have two RAPPA variants

Fifty-one patients (6%) carried at least one rare, additional, potentially protein-altering variant. A summary of all the different rare, additional, potentially protein-altering variants, along with their gnomAD frequency, and count in this dataset is given in Supplementary Table S1.

### Impact of rare, additional, potentially protein-altering variants on age at onset of kidney failure

The impact of rare, additional, potentially protein-altering variants on kidney survival was then assessed using a multivariable Cox model (see Methods). Individuals who carried rare, additional, potentially protein-altering variants in *PKD1* were more likely to progress to kidney failure relative to patients without such variants (Hazard ratio (HR) = 1.66; 95% Confidence Interval (CI): 1.18–2.34; *P* = 0.003; Table 2). The variance explained by this model was 0.20, compared to 0.19 for the model with just sex and diagnostic variant type as predictors. Those with rare, additional, potentially protein-altering variants developed kidney failure on average four years earlier than those without rare, additional, potentially protein-altering variants (*P* = 0.009; Supplementary Figure S1).

**Table 2:** The impact of RAPPA variants on kidney survival, age at first urological event, and age at hypertension diagnosis. Multivariable Cox model controlling for type of ADPKD diagnostic variant and sex

Characteristic	HR (95% CI)	*p*-value
**Kidney survival (*n* = 932, *R***^**2**^** = 0.20)**		
Diagnostic variant type		
PKD1-T	1	–
PKD1-NT	0.41 (0.33, 0.49)	** < 0.001**
Sex		
Female	1	–
Male	1.53 (1.28, 1.83)	** < 0.001**
**RAPPA variant (*n*** **= 51)**	**1.66 (1.18, 2.34)**	**0.003**
**Age at first urological event (*n*** **= 323, *R***^**2**^ **= 0.02)**		
Diagnostic variant type		
PKD1-T	1	–
PKD1-NT	0.87 (0.66, 1.15)	0.34
Sex		
Female	1	–
Male	1.23 (0.95, 1.60)	0.12
**RAPPA variant (*n* = 17)**	1.65 (0.94, 2.90)	0.08
**Age at hypertension diagnosis (*n* = 316,** ***R***^**2**^ **= 0.02)**		
Diagnostic variant type		
PKD1-T	1	–
PKD1-NT	0.82 (0.63, 1.06)	0.13
Sex		
Female	1	–
Male	1.09 (0.85, 1.38)	0.51
**RAPPA variant (*n*** **= 17)**	1.58 (0.93, 2.66)	0.08

*p*-values less than 0.05 are bolded

*CI* Confidence Interva, *HR* Hazard ratiol, *RAPPA* Rare, Additional, Potentially Protein Altering (RAPPA) *PKD1* variants, *PKD1*-NT *PKD1*-non truncating variant, *PKD1*-T *PKD1* truncating variant

To investigate whether some of the observed effect of rare, additional, potentially protein-altering variants on age at kidney failure was driven by relatedness between individuals, a Cox model for the impact of rare, additional, potentially protein-altering variants was run on a subset of 747 unrelated individuals. The impact of rare, additional, potentially protein-altering variants on kidney survival was consistent in this cohort of unrelated individuals (HR: 1.55; 95% CI 1.05–2.25; *P* = 0.03; Supplementary Table S2), relative to the full cohort (HR: 1.66; 95% CI 1.18–2.34;* P* = 0.003).

### Incorporating phasing information with rare, additional, potentially protein-altering variants

We next examined the relevance of phase to age at kidney failure, focusing on the subset of rare, additional, potentially protein-altering variants that were *in trans* with the diagnostic variants. As a result of SHAPEIT being unable to calculate phase for singletons with the required level of accuracy[18] (phasing confidence greater than 0.99[18]), computational phasing data were only successfully obtained on one individual. All other phasing data were obtained based on familial phasing information. We were able to determine whether a variant was *in cis* or *in trans* with the diagnostic variant for 20 of the 51 patients with rare, additional, potentially protein-altering variants. Individuals with *trans* rare, additional, potentially protein-altering variants (*n* = 13/894) had a greater risk of kidney failure compared to those without (HR: 1.83; 95% CI 1.00–3.33; *P* = 0.049; Table 3).

**Table 3:** The impact of *in trans* RAPPA variants on kidney survival

Characteristic	HR (95% CI)	*p*-value
**Kidney survival (*n* = 894, *R***^**2**^ **= 0.20)**		
Diagnostic variant type		
PKD1-T	1	**-**
PKD1-NT	0.40 (0.33, 0.49)	** < 0.001**
Sex		
Female	1	**-**
Male	1.56 (1.30, 1.87)	** < 0.001**
***In trans*** **RAPPA variant (*n* = 13)**	**1.83 (1.00, 3.33)**	**0.049**

*p*-values less than 0.05 are bolded

*CI* Confidence Interva, *HR* Hazard ratiol, *RAPPA* Rare, Additional, Potentially Protein Altering (RAPPA) *PKD1* variants, *PKD1*-NT *PKD1*-non truncating variant, *PKD1*-T *PKD1* truncating variant

As expected, individuals with rare, additional, potentially protein-altering variants *in cis* with the diagnostic variant (*n* = 7) did not have a significantly different risk of kidney failure than those without any rare, additional, potentially protein-altering variants (HR = 1.59, 95% CI 0.51–4.98, *P* = 0.42).

### Impact of rare, additional, potentially protein-altering variants on other kidney phenotypes

We next investigated the association of rare, additional, potentially protein-altering variants on both age at first urological event and age at hypertension diagnosis, using multivariable models (see Methods). The presence of rare, additional, potentially protein-altering variants trended towards increased risk for both age at first urological event (HR: 1.65; 95% CI 0.94–2.90; *P* = 0.08) and age at hypertension diagnosis (HR: 1.58; 95% CI 0.93–2.66; *P* = 0.08; Table 1), but neither was significantly associated, possibly due to inadequate power and resulting from the relatively small group sizes.

Finally, we investigated the effect of rare, additional, potentially protein-altering variants on htTKV, MIC, and PROPKD score using a series of linear models (see Methods). The presence of rare, additional, potentially protein-altering variants was not significantly associated with any of these outcomes (*P* = 0.24, 0.33, and 0.33 respectively, Table 4).

**Table 4:** The impact of RAPPA variants on log transformed htTKV, PROPKD score, and MIC score. Multivariable linear model controlling for age at imaging, type of ADPKD diagnostic variant, and sex

Characteristic	Beta (95% CI)	*p*-value
**Log htTKV (*n* = 379,** ***R***^**2**^** = 0.21)**		
Intercept	6.46 (6.16, 6.77)	** < 0.001**
Age at imaging	0.008 (0.002, 0.014)	**0.009**
Diagnostic variant type		
PKD1-T	0	–
PKD1-NT	– 0.34 (– 0.50, – 0.19)	** < 0.001**
Sex		
Female	0	–
Male	0.70 (0.54, 0.85)	** < 0.001**
**RAPPA variant (*n* = 14)**	– 0.24 (– 0.63, 0.16)	0.24
**PROPKD score (1–9) (*n*** ***= 328,*** ***R***^**2**^ **= 0.003)**		
Intercept	5.5 (5.3, 5.7)	** < 0.001**
**RAPPA variant (*n*** **= 21)**	0.42 (– 0.42, 1.27)	0.33
**MIC score (1–5) (*n*** **= 382,** ***R***^**2**^** = 0.003)**		
Intercept	3.33 (3.20, 3.46)	** < 0.001**
**RAPPA variant (***n*** = 14)**	– 0.33 (– 1.00, 0.33)	0.33

*p*-values less than 0.05 are bolded

*CI* Confidence Interval, *RAPPA* Rare, Additional, Potentially Protein Altering (RAPPA) *PKD1* variants, *PKD1*-NT *PKD1*-non truncating variant, *PKD1*-T *PKD1* truncating variant

## Discussion

Identifying clinical and genetic predictors of ADPKD disease progression is important to enable clinical decisions, especially in light of the approval of disease-modifying therapies like tolvaptan. In this study, we attempted to redefine the role of *PKD1* rare, additional, potentially protein-altering variants in influencing ADPKD phenotype in the context of a genetically-confirmed ADPKD diagnosis. Analysing a sample of 932 genetically-defined ADPKD patients assembled across seven cohorts, we identified a subset of variants of unknown significance which we have termed “ rare, additional, potentially protein-altering variants” that were likely to be protein-altering, and thus potentially influencing the phenotype. Our results suggest rare, additional, potentially protein-altering variants have a detrimental effect on disease progression. Patients who carry rare, additional, potentially protein-altering variants reached kidney failure on average 4 years earlier than non-carriers, and had a 68% greater risk of progression to kidney failure. These results are consistent with the hypothesis that rare, additional, potentially protein-altering variants have an adverse impact on modulating levels of functional PC1 protein.

Clinically, the European Renal Association − European Dialysis and Transplant Association (ERA-EDTA) consensus statement recommends evaluating ADPKD patients' risk of rapid progression based on estimated GFR (eGFR) decline, TKV increase rate, and other factors like genotype and PROPKD score[19]. Our analyses suggest that rare, additional, potentially protein-altering variants may be an additional factor to consider when evaluating ADPKD progression risk. This is the first evidence generated using a robust statistical framework across multiple study sites suggesting that rare, additional, potentially protein-altering *PKD1* variants have a phenotypic impact in influencing disease progression in genetically-confirmed ADPKD. This study provides suggestive evidence that rare, additional, potentially protein-altering variants in *PKD1* can improve ADPKD disease progression prediction, an important concern for ADPKD patients and physicians, at a population level across cohorts.

Studies from other investigators have demonstrated the role of rare variation in patients' genetic background in modulating disease severity[7, 20–26]. One study found that 14% of their 174 patients with ADPKD also carried additional rare variants in the *PKD1* gene, which are predicted to be detrimental to protein formation[9]. The filtering criteria used by Audrézet et al. were more relaxed than the criteria we applied[9]. Notable differences include that Audrézet et al. also looked at rare variants in *PKD2*, *PKHD1,* and *HNF1B,* whereas our study focused on rare variants in *PKD1*, employing combined annotation-dependent depletion and rare exome variant ensemble learner scores for filtering in addition to ACMG classification. It is thus not surprising that different prevalences of rare variants were observed between the two studies (6% prevalence of rare, additional, potentially protein-altering variants in our study). However, the Audrézet study did not compare phenotypic outcomes between those with such variants and those without[9]. Whilst we did not examine the precise mechanism of action of rare, additional, potentially protein-altering variants, a rare, additional, potentially protein-altering in *PKD1* may act as a hypomorphic variant, slightly altering the structure of one copy of polycystin 1 or reducing its ability to interact with the remaining functional polycystin 2.

Although we have focused on the phenotype modifying role of *PKD1* variants, our results motivate further consideration of variants that have been labelled variants of unknown significance and their impact on the phenotype. Future work could investigate the role of rare variant burden analysis in other genes implicated in PKD, and other forms of inherited kidney and other diseases. There is emerging evidence that rare, predicted damaging variants within cystic kidney disease genes such as *PKD2, HNF1B* and *PKHD1* may have a phenotype modifying role[6, 7, 27, 28]. Further understanding of this genetic risk can impact the care provided to individuals in this high-risk population for kidney failure, including patient counselling, and increased disease monitoring.

We note that variation in disease severity is a topic of much discussion in ADPKD but also in other forms of inherited kidney disease such as autosomal dominant tubulointerstitial kidney disease (ADTKD) and Alport syndrome. Specific Alport syndrome pathogenic variants in *COL4A3* and *COL4A4* can cause sub-clinical phenotypes in some patients, and severe manifestations of disease in others[29]. The *UMOD* NM_003361.4:p.(T62P) variant has recently been described as a variant which may infer risk for ADTKD but is present at a relatively high frequency in control populations[30]. The rare, additional, potentially protein-altering variants studied here are conceptually distinct from these risk alleles and variants with incomplete penetrance in that they are not likely to cause disease in isolation, but may provide an explanation for such variation in disease severity.

There are limitations to our study. We were unable to determine phase for most of our cohort due to the limitations imposed by short-read sequencing, computational phasing tools and the lack of parental (or additional familial) genotype. This prevented in-depth analysis of any *cis* or *trans* signals. The computational phasing tools we used provide estimates of variant phasing and may not be as reliable as other phasing methods such as familial segregation analysis or long-read sequencing, which for practical reasons, were unavailable to this study. Centralised variant calling was not possible due to data sharing restrictions across the multiple study cohorts, although the same standardised variant filtering criteria were used by all centres and all summary-level data were reviewed and analysed centrally. Given that some of the rare, additional, potentially protein-altering variants are relatively common ( minor allele frequency up to 1%), it is possible that the functionality of some rare, additional, potentially protein-altering variants occurs through an alternative variant in complete linkage disequilibrium with the rare, additional, potentially protein-altering variant.

We were unable to detect a significant association between rare, additional, potentially protein-altering variants and several of the phenotypes of interest including htTKV and PROPKD score. This is possibly due to a lack of power as these data were only available for a relatively small proportion of our cohort (41% and 35%, respectively). The *PKD1* gene is particularly complex with several pseudogenes and regions of high GC content, and so it is possible that some rare, additional, potentially protein-altering variants were missed as part of the variant identification process.

In summary, we provide novel insights into the relationship between genetic burden and kidney disease severity. Further study of such rare, additional, potentially protein-altering variants in larger cohorts with deeper phenotyping will be required before considering clinical implementation.

## Supplementary Information

Below is the link to the electronic supplementary material.

Supplementary file1 (DOCX 64 KB)

## Data Availability

The data used in this study are not publicly available due to concerns regarding patient confidentiality and privacy. Access to the data can be requested from each participating centre individually, subject to their respective data sharing policies and ethical considerations.
