# Supplementary material for: The impact of a secondary, rare, non-pathogenic PKD1 variant on disease progression in autosomal dominant polycystic kidney disease
Source: J Nephrol. 2025 Jan 30;38(5):1457–67. doi: 10.1007/s40620-025-02211-x (PMC12289752; doi:10.1007/s40620-025-02211-x)
Supplement: Supplementary file 1 — Supplementary file1 (DOCX 64 KB) [file 40620_2025_2211_MOESM1_ESM.docx]

**Supplementary Material**

**Table of contents**

[Detailed patient and variant information 2](#_gjdgxs)

[**Familial analysis 4**](#_k9fgqz7waqxq)

# Details of centres involved

| **Study** | **Reference publication** |
| --- | --- |
| Dublin-Ireland (Beaumont Hospital) | Benson et al 2021[[1]](https://www.zotero.org/google-docs/?7BaxSK) |
| Leipzig-Germany (University of Leipzig Medical Center) | Schӧnhauer et al 2020[[2]](https://www.zotero.org/google-docs/?chwOwj) |
| Bologna-Italy (Alma Mater Studiorum, University of Bologna) | Mantovani et al 2020[[3]](https://www.zotero.org/google-docs/?dfZ9ft) |
| Sydney-Australia (Garvan Institute of Medical Research) | Mallawaarachchi et at 2016[[4]](https://www.zotero.org/google-docs/?OF9DQ2) |
| Nantes-France (Nantes University Hospital) | Currently unpublished |
| Rochester-United States (Mayo Clinic) | Mallawaarachchi et al 2021[[5]](https://www.zotero.org/google-docs/?km5WY6) |
| London-United Kingdom (Genomics England) | The National Genomic Research library 2020[[6]](https://www.zotero.org/google-docs/?e4BsHV) |

# Detailed patient and variant information

**Supplementary Table S1:** Summary of the RAPPA variants in this cohort. The ACMG classification of each variant, along with its global gnomAD frequency and count in this dataset are also given.

| **RAPPA** | **ACMG Class** | **gnomAD frequency** | **Count** |
| --- | --- | --- | --- |
| p.Arg1547His | VUS (PM2) | 0.00001924 | 1 |
| p.Arg3647Trp | VUS (PM2, BS2) | 0.0004605 | 2 |
| p.Ser1957Arg | VUS (PM2) | 0 | 1 |
| p.Gln1908Pro | VUS (PM2) | 0.0000012 | 1 |
| p.Arg3748Trp | VUS (PM2, PP3) | 0.0000006853 | 1 |
| p.Pro450Thr | VUS (PM2, BP4) | 0.0001805 | 2 |
| p.Arg2186Cys | VUS (PM2) | 0.00001732 | 1 |
| p.Cys874Phe | VUS (PM2) | 0.0000012 | 1 |
| p.Thr775Met | VUS (PM2) | 0.00001302 | 1 |
| p.Leu3477Ile | VUS (PM2, BP4) | 0.0003457 | 2 |
| p.Ser2807Tyr | VUS (PM2) | 0 | 1 |
| p.Leu1442Phe | VUS (PM2) | 0.00000137 | 1 |
| p.Pro398Leu | VUS (PM2, PP3, PP5) | 0.00003721 | 1 |
| p.Arg2220Gly | VUS (PM2, PM5) | 0.000008703 | 1 |
| p.Asp2972Asn | VUS (PM2, BP4) | 0.0001312 | 1 |
| p.His2998Gln | VUS (PM2) | 0.000001371 | 1 |
| p.Thr1589Met | VUS (PM2) | 0.000006848 | 1 |
| p.Arg2024Cys | VUS (PM2, PP3) | 0.0000237 | 2 |
| p.Gly3877Ser | VUS (PM2) | 0.000009066 | 2 |
| p.Pro655Leu | VUS (PM2, BP1) | 0.0001294 | 1 |
| p.Arg3247Cys | VUS (PM2, PM5, PP3) | 0.00001133 | 1 |
| p.Gly1944Arg | VUS (PM2, PP3) | 0 | 1 |
| p.Ala989Thr | VUS (PM2) | 0.00000809 | 2 |
| p.Ala1985Thr | VUS (PM2) | 0.000002081 | 2 |
| p.Asp1246Asn | VUS (PM2) | 0.00003288 | 1 |
| p.Ala2871Thr | VUS (PM2) | 0.00009129 | 1 |
| p.Lys3607Met | VUS (PM2, PP3) | 0 | 2 |
| p.Val49Leu | VUS (PM2, BP4) | 0 | 1 |
| p.Gly60Cys | VUS (PM2) | 0 | 1 |
| p.Ile2646Thr | VUS (PM2, BS2) | 0.0002447 | 1 |
| p.Glu2810Lys | VUS (PM2, BP4) | 0.0001068 | 1 |
| p.Thr1354Met | VUS (PM2) | 0.0000447 | 1 |
| p.Glu3925Ala | VUS (PM2) | 0.000004691 | 1 |
| p.Gly2452Cys | VUS (PM2, PM1) | 0 | 1 |
| p.Ala3991Val | VUS (PM2) | 0.00002894 | 1 |
| p.Arg2518His | VUS (PM2) | 0.00002298 | 1 |
| p.Arg3209Cys | VUS (PM2, BP1) | 0.00004594 | 1 |
| p.Asp3239Asn | VUS (PM2, PP3) | 0.00001374 | 1 |
| p.Asn2620Ser | VUS (PM2, PP3) | 0.0002506 | 1 |
| p.Arg3348Gln | VUS (PM2, BS2) | 0.009045 | 1 |
| p.Met2760Thr | VUS (PM2) | 0.000001864 | 1 |
| p.Glu685Gly | VUS (PM2, PM5) | 0.0000007005 | 1 |
| p.Pro2965Ala | VUS (PM2, BP4) | 0 | 1 |
| p.Gln2670Arg | VUS (PM2, BS4) | 0.0002911 | 1 |
| p.Arg1425Cys | VUS (PM2, BP4) | 0.00002358 | 1 |
| p.Arg600Trp | VUS (PM2, BP4) | 0.00003548 | 1 |

RAPPA: Rare, Additional, Potentially Protein Altering (RAPPA) VUS: variant of unknown significance

# Familial analysis

**Supplementary Table S2.** The impact of RAPPA variants on kidney survival, taking just one individual from each family. Cox model controlling for diagnostic variant type and sex.

| **Characteristic** | **HR (95% CI)** | ***P*-value** |
| --- | --- | --- |
| **Kidney survival (n = 747, R^2^ = 0.22)** | | |
| Diagnostic variant type |  |  |
| *PKD1-T* | 1 | **-** |
| *PKD1-NT* | 0.38 (0.30, 0.47) | **<0.001** |
| Sex |  |  |
| *Female* | 1 | **-** |
| *Male* | 1.52 (1.24, 1.85) | **<0.001** |
| **RAPPA variant (n = 42)** | **1.55 (1.05, 2.25)** | **0.03** |

CI: Confidence Interval, HR: Hazard ratio, RAPPA: Rare, Additional, Potentially Protein Altering (RAPPA) *PKD1* variants, *PKD1*-NT: *PKD1*-non truncating mutation, *PKD1*-T: *PKD1* truncating mutation.

# Survival curves for individuals with and without RAPPA variants


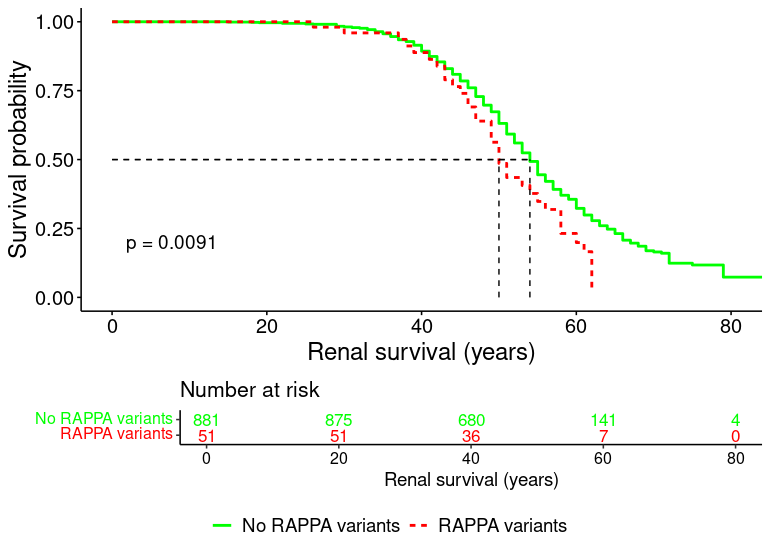


**Supplementary Figure S1:** Kaplan-Meier plot comparing kidney survival of patients with and without RAPPA variants. Dotted lines indicate median survival for each group. With RAPPA variants n=51, without n=881. Median survival of those without RAPPA variants is 54 years, median survival of those with RAPPA variants is 50 years (*P* = 0.009).

#

# References

[[1] K. A. Benson *et al.*, “The genetic landscape of polycystic kidney disease in Ireland,” *European Journal of Human Genetics*, vol. 29, no. 5, pp. 827–838, 2021, doi: 10.1038/s41431-020-00806-5.](https://www.zotero.org/google-docs/?9Adamk)

[[2] R. Schönauer *et al.*, “Matching clinical and genetic diagnoses in autosomal dominant polycystic kidney disease reveals novel phenocopies and potential candidate genes,” *Genetics in Medicine*, vol. 22, no. 8, pp. 1374–1383, Aug. 2020, doi: 10.1038/s41436-020-0816-3.](https://www.zotero.org/google-docs/?9Adamk)

[[3] V. Mantovani *et al.*, “Gene Panel Analysis in a Large Cohort of Patients With Autosomal Dominant Polycystic Kidney Disease Allows the Identification of 80 Potentially Causative Novel Variants and the Characterization of a Complex Genetic Architecture in a Subset of Families,” *Frontiers in Genetics*, vol. 11, no. May, pp. 1–14, May 2020, doi: 10.3389/fgene.2020.00464.](https://www.zotero.org/google-docs/?9Adamk)

[[4] A. C. Mallawaarachchi *et al.*, “Whole-genome sequencing overcomes pseudogene homology to diagnose autosomal dominant polycystic kidney disease,” *European Journal of Human Genetics*, vol. 24, no. 11, pp. 1584–1590, Nov. 2016, doi: 10.1038/ejhg.2016.48.](https://www.zotero.org/google-docs/?9Adamk)

[[5] A. C. Mallawaarachchi *et al.*, “Genomic diagnostics in polycystic kidney disease: an assessment of real-world use of whole-genome sequencing,” *European Journal of Human Genetics*, pp. 1–11, Jan. 2021, doi: 10.1038/s41431-020-00796-4.](https://www.zotero.org/google-docs/?9Adamk)

[[6] Genomics England, “The National Genomic Research Library v5.1.”](https://www.zotero.org/google-docs/?9Adamk)
